# Delayed malposition of a double-lumen hemodialysis catheter that caused hemorrhage and hypovolemic shock: A case report

**DOI:** 10.1097/MD.0000000000014192

**Published:** 2019-01-18

**Authors:** I-Chen Chen, Shih-Chia Yang, Kuan-Ting Liu, Yen-Hung Wu

**Affiliations:** a Department of Emergency Medicine, Kaohsiung Medical University Hospital; b School of Medicine, College of Medicine, Kaohsiung Medical University, Kaohsiung, Taiwan.

**Keywords:** dislocation, double-lumen catheter, hemorrhage, shock

## Abstract

**Rationale:**

Double-lumen hemodialysis catheters are commonly used as temporary hemodialysis routes. Complications include infection, thrombosis, cardiac arrhythmia, entrapped guide wire, and malposition. We report a rare complication of delayed hemodialysis catheter malposition that caused retroperitoneal hemorrhage and hypovolemic shock during hemodialysis.

**Patient concerns:**

A 72-year-old female patient who was receiving hemodialysis was referred to our emergency department because of general discomfort and decreased blood pressure (BP) after her regular hemodialysis. She had undergone surgery for a left forearm arteriovenous pseudoaneurysm and received a temporary hemodialysis catheter insertion via the left femoral vein 2 weeks before. The initial blood examination revealed a mildly decreased baseline hemoglobin level (7.2 g/dL) and hyperkalemia (5.9 mmol/L). Her BP recovered after fluid resuscitation. She was administered hemodialysis again, following which her BP reduced and a change in consciousness developed.

**Diagnosis:**

Chest and abdominal computed tomographies were performed to exclude acute vascular problems and showed a hemodialysis catheter tip protruding from the left iliac vein and hematoma in the left retroperitoneal space and pelvic cavity.

**Interventions:**

Intubation, fluid resuscitation, vasopressor administration, and blood transfusion were performed. She was admitted to the intensive care unit. The left femoral hemodialysis catheter was removed.

**Outcomes:**

Follow-up computed tomography revealed resolution of the retroperitoneal space hematoma. She was transferred to the ordinary ward 18 days later with a stable hemodynamic status. Unfortunately, she developed hospital-acquired pneumonia and arteriovenous shunt infection, and died from respiratory failure and sepsis on the 34th day in our hospital.

**Lessons:**

Femoral double-lumen catheter malposition is rare and potentially fatal. Emergency physicians should be aware of situations wherein a patient's BP declines markedly soon after a hemodialysis initiation.

## 1 Introduction

Double-lumen hemodialysis catheters are commonly used as temporary hemodialysis routes. Complications of central venous hemodialysis catheters include infection, thrombosis, cardiac arrhythmia, entrapped guide wire, and malposition.^[1]^ Malposition is usually found as an acute complication. We report a rare complication of delayed hemodialysis catheter malposition that caused retroperitoneal hemorrhage and hypovolemic shock. After we consulted the regulations of the institutional review board of the Kaohsiung Medical University Hospital, ethical approval for this case report was not required. Informed consent was obtained from the patient's family.

## 2 Case presentation

**Figure 1:**
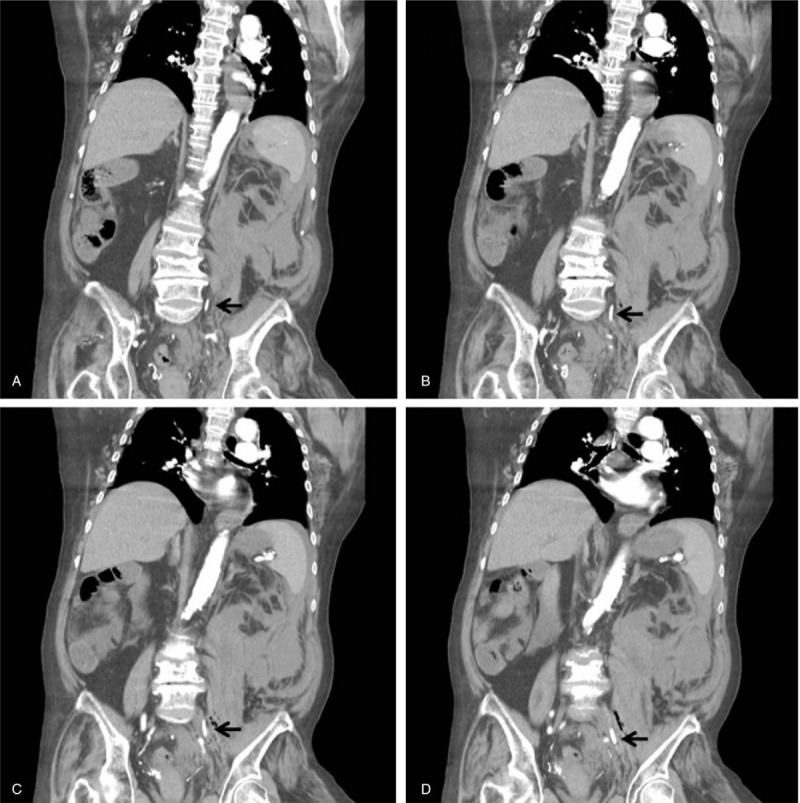
Computed tomography image of the patient showing a double-lumen catheter tip protruding from the left iliac vein (arrow) and hematoma in the retroperitoneal space and pelvic cavity.

A 72-year-old woman was referred to our emergency department from a dialysis clinic because of general discomfort and decreased blood pressure within 30 min after hemodialysis was initiated. Her status on arrival was as follows: blood pressure, 85/54 mmHg; heart rate, 122 bpm; and body temperature, 35.8°C. Her consciousness was clear. She complained of general discomfort, but no chest or abdominal pain was observed. Physical examination revealed a pale conjunctiva and soft abdomen. No bloody/tarry stool passage was observed. She had diabetes mellitus and uremia and underwent regular hemodialysis. She had undergone surgery for a left forearm arteriovenous pseudoaneurysm 15 days before, and a temporary hemodialysis catheter was inserted via the left femoral vein on the day after surgery. She had undergone hemodialysis via a left femoral catheter several times without any problems until this time. Blood examination revealed the following values: white blood cells, 27,580/μL; hemoglobin, 7.2 g/dL; platelets, 269,000/μL; glucose, 347 mg/dl; glutamic oxaloacetic transaminase/glutamic pyruvic transaminase, 29/10 IU/L; C-reactive protein, 13.36 mg/L; sodium, 136 mmol/L; potassium, 5.9 mmol/L; creatine phosphokinase, 78 IU/L; creatine kinase-muscle/brain, 5.3 ng/mL; troponin I, 0.039 ng/mL; pH, 7.114; and HCO_3_^−^, 13.8 mmol/L. Her blood pressure recovered after normal saline hydration and packed red blood cell transfusion. Owing to the incomplete regular hemodialysis and hyperkalemia, she was referred to the hemodialysis department to undergo hemodialysis again. Reduced blood pressure and change in consciousness developed soon after hemodialysis was initiated. She was intubated and referred back to the emergency department. Subsequently, central venous catheter insertion, fluid resuscitation, and vasopressor administration were performed. Blood examinations were repeated. Computed tomography was performed to exclude vascular emergency and revealed a double-lumen catheter tip protruding from the left iliac vein and hematoma in the left retroperitoneal space and pelvic cavity (Fig. 1). Blood examination results revealed the following values: white blood cells, 26,840/μL; hemoglobin, 3.9 g/dL; platelets, 182,000/μL; pH, 7.018; HCO_3_^−^, 10.2 mmol/L; lipase 64 U/L; creatine phosphokinase, 152 IU/L; creatine kinase-muscle/brain, 9.6 ng/mL; troponin I, 0.106 ng/mL; and lactate, 11.5 mmol/L. She was then admitted to the intensive care unit for further care. The left femoral hemodialysis catheter was removed, and follow-up computed tomography revealed resolution of the retroperitoneal space hematoma. She was transferred to the ordinary ward 18 days later with stable hemodynamic status. Unfortunately, the patient developed hospital-acquired pneumonia and suspected arteriovenous shunt infection, and died from respiratory failure and sepsis on the 34th day after being transferred to our hospital.

## 3 Discussion

The complications of using hemodialysis catheters include infection, thrombosis, hemorrhage, cardiac arrhythmia, entrapped guide wire, and malposition.^[1]^ Some of these complications can be fatal.^[2,3]^ Malposition of the jugular or subclavian double-lumen central catheter is not an uncommon complication.^[3–5]^ Femoral double-lumen central venous hemodialysis catheter malposition in adults is rare,^[6–8]^ and most cases are acute, occurring right after the catheter insertion, but delayed malposition was found in our case.

Tsai et al reported a case of shock after initiating hemodialysis due to the malposition of a femoral double lumen catheter.^[6]^ The proximal opening was in the vessel, but not the distal opening. Shock developed when hemodialysis was initiated, during which the machine drained blood from the patient via the proximal opening directly and injected the blood into the patient's retroperitoneum via the distal opening. The condition was just like that of our patient, except that they used the catheter for the first time. Malposition is usually an acute complication. Our patient had received hemodialysis via a double-lumen catheter several times smoothly in the past; this made the diagnosis more difficult. Haid et al also reported a dislocation of a double-lumen tunneled hemodialysis catheter that caused dysfunction and bleeding.^[9]^ Their case involved malposition of a catheter after it was pulled out. The proximal limb opening was outside the vessel, and the distal limb opening was still in the internal jugular vein. As no blood could be aspirated from the proximal arterial limbs, the 2 catheter limbs were connected in an inverse fashion. Infraclavicular hematoma developed soon after hemodialysis was initiated. Hemodialysis catheter malposition with one opening inside the vessel and another opening outside the vessel may have caused severe bleeding, as blood was drawn from the opening inside the vessel and injected in the opening outside the vessel.

Although the reason for delayed malposition in this patient was unclear, we thought that the most probable reason for this was displacement during movement of the left lower extremity, as the patient was completely independent. Malposition of femoral double-lumen catheters is rare and potentially fatal. Emergency physicians should be aware of situations wherein a patient's blood pressure declines markedly soon after hemodialysis initiation.

## Author contributions

**Resources:** I-Chen Chen, Kuan-Ting Liu.

**Supervision:** Shih-Chia Yang.

**Visualization:** Shih-Chia Yang, Kuan-Ting Liu, Yen-Hung Wu.

**Writing – original draft:** I-Chen Chen.

**Writing – review & editing:** Yen-Hung Wu.
